# Clinical efficacy of avatrombopag and recombinant human thrombopoietin in the treatment of chronic liver disease-associated severe thrombocytopenia: A real-world study

**DOI:** 10.3389/fphar.2022.1009612

**Published:** 2022-10-04

**Authors:** Yong-Shuai Wang, Wei Wang, Sai Zhang, Shen-Yu Zhang, Ai-Zong Shen, Wei Wang, Hua-Chuan Song, Huan-Zhang Yao, Rui-Peng Song, Fan-Zheng Meng, Lei Li, Bjoern Nashan, Ji-Zhou Wang, Lian-Xin Liu

**Affiliations:** ^1^ Department of Hepatobiliary Surgery, The First Affiliated Hospital of USTC, Division of Life Sciences and Medicine, University of Science and Technology of China, Hefei, Anhui, China; ^2^ Department of Medical Oncology, The First Affiliated Hospital of USTC, Division of Life Sciences and Medicine, University of Science and Technology of China, Hefei, Anhui, China; ^3^ Department of Pharmacy, The First Affiliated Hospital of USTC, Division of Life Sciences and Medicine, University of Science and Technology of China, Hefei, Anhui, China; ^4^ Department of Pathology, The First Affiliated Hospital of USTC, Division of Life Sciences and Medicine, University of Science and Technology of China, Hefei, Anhui, China; ^5^ Department of Infectious Disease, The First Affiliated Hospital of USTC, Division of Life Sciences and Medicine, University of Science and Technology of China, Hefei, Anhui, China; ^6^ Department of Organ transplant center, The First Affiliated Hospital of USTC, Division of Life Sciences and Medicine, University of Science and Technology of China, Hefei, Anhui, China; ^7^ Anhui Province Key Laboratory of Hepatopancreatobiliary Surgery, Hefei, Anhui, China

**Keywords:** avatrombopag, thrombocytopenia (TCP), chronic liver disease (CLD), recombinant human thrombopoietin (rh-TPO), propensity scorematching (PSM), efficacy

## Abstract

**Purpose:** To investigate the clinical efficacy of avatrombopag, an oral thrombopoietin receptor agonist, versus subcutaneous recombinant human thrombopoietin (rh-TPO) in the treatment of severe thrombocytopenia (TCP) associated with chronic liver disease (CLD).

**Methods:** Clinical data of 250 patients with severe TCP associated with CLD were collected in a single hospital from January 2019 to January 2022. The main parameters measured were the therapeutic response rate, changes in platelets (PLTs), and adverse events. Propensity score matching (PSM) was used to avoid possible selection bias.

**Results:** After PSM, a total of 154 patients were enrolled in the study: 77 in the avatrombopag group and 77 in the rh-TPO group. There was no statistically significant difference between the two groups in the effect of increasing the PLT count (*Waldχ* ^*2*^ = 1.659, *p* = 0.198; *Waldχ* ^*2*^ = 0.220, *p* = 0.639). In addition, no interaction between time and different medications was found (*Waldχ* ^*2*^ = 0.540, *p* = 0.910; *Waldχ* ^*2*^ = 1.273, *p* = 0.736). Interestingly, in the subgroup analysis, both before and after PSM, avatrombopag showed better clinical efficacy than rh-TPO in the treatment of TCP associated with CLD in Child‒Pugh Class A (88.89% vs. 63.41%, *p =*0.003; 81.33% vs*.* 61.76%, *p* = 0.043). Fewer patients reported dizziness in the avatrombopag group than in the rh-TPO group both before and after PSM (7.8% vs. 25.0%; 7.8% vs. 24.7%, *p* < 0.05).

**Conclusion:** Both before and after PSM, avatrombopag showed better clinical efficacy than rh-TPO in the treatment of TCP associated with CLD in Child‒Pugh Class A and showed a lower incidence of dizziness in all patients.

## 1 Introduction

Thrombocytopenia (TCP) is the most common hematological complication in patients with chronic liver disease (CLD) (Qamar et al., 2009). Due to the decrease in the platelet count, these patients usually have a higher risk of bleeding during invasive procedures, resulting in increased postoperative complications and mortality (Giannini et al., 2010; Forkin et al., 2018). Traditionally, a growing number of studies have been suggesting that prophylactic platelet (PLT) transfusion before or after invasive procedures can improve the hemostatic potential and reduce the risk of bleeding in CLD patients with severe TCP (platelet count <50 × 10^9^/L) (Maan et al., 2015; Mitchell et al., 2016). This threshold is used as a standard for clinical care and invasive procedures (Scharf, 2021).

Thrombopoietin (TPO) is an important physiological regulator of thrombopoiesis and megakaryocyte maturation, which increases the number of PLTs in the blood by stimulating the production of endogenous functional PLTs. Accumulating studies have revealed that TPO is a therapeutic target that stimulates PLT production (Qureshi et al., 2016; Gilreath et al., 2021).

When considering cost-effectiveness, analysis of increasing PLT values to the same level using traditional PLT infusion may be less costly than TPO agonists. Many centers still use traditional PLT transfusion. However, PLT transfusion may be associated with serious complications, including transfusion reactions and the transmission of infectious agents, which can be fatal in rare cases, and alloimmunization can occur in patients who have received multiple blood product transfusions because recipients form antibodies against donor platelets (Basili et al., 2018; Armstrong et al., 2020; Elsaid et al., 2020; Karlström et al., 2022). In addition, platelet transfusions have only a short-term effect, while TPO agonists have a longer effect. Thus, new and efficient drugs targeting this issue need to be developed and explored in clinical practice.

Recombinant human thrombopoietin (rh-TPO) is generally used for treating thrombocytopenia caused by chemotherapy and immune thrombocytopenia (Vadhan-Raj et al., 2003; Yu et al., 2020). There are no prospective clinical trials to confirm its use in CLD, but multicenter real-world studies have demonstrated its efficacy and safety in CLD patients with TPO (Feng et al., 2022). Avatrombopag, as a novel oral TPO receptor agonist, has been approved recently as an alternative treatment for PLT in the treatment of invasive surgery for CLD patients with severe TCP. Phases I, II, and III studies demonstrated the efficacy and safety of avatrombopag in patients with severe TCP related to CLD (Terrault et al., 2014; Kuter and Allen, 2018; Terrault et al., 2018). Since both drugs are used in this condition, a subsequent issue arises: whether the two drugs can increase platelet count before invasive surgical procedures, whether oral and subcutaneous injections have the same efficacy, and whether there is a difference in adverse drug events effects. However, to date, few studies have focused on the comparison between the efficacy and safety of avatrombopag and rh-TPO.

Thus, in the present study, we analyzed the clinical data of patients with TCP associated with CLD before and after propensity score matching (PSM) to explore the efficacy of avatrombopag compared with rh-TPO in the real-world study.

## 2 Methods and materials

### 2.1 Patients

**FIGURE 1:**
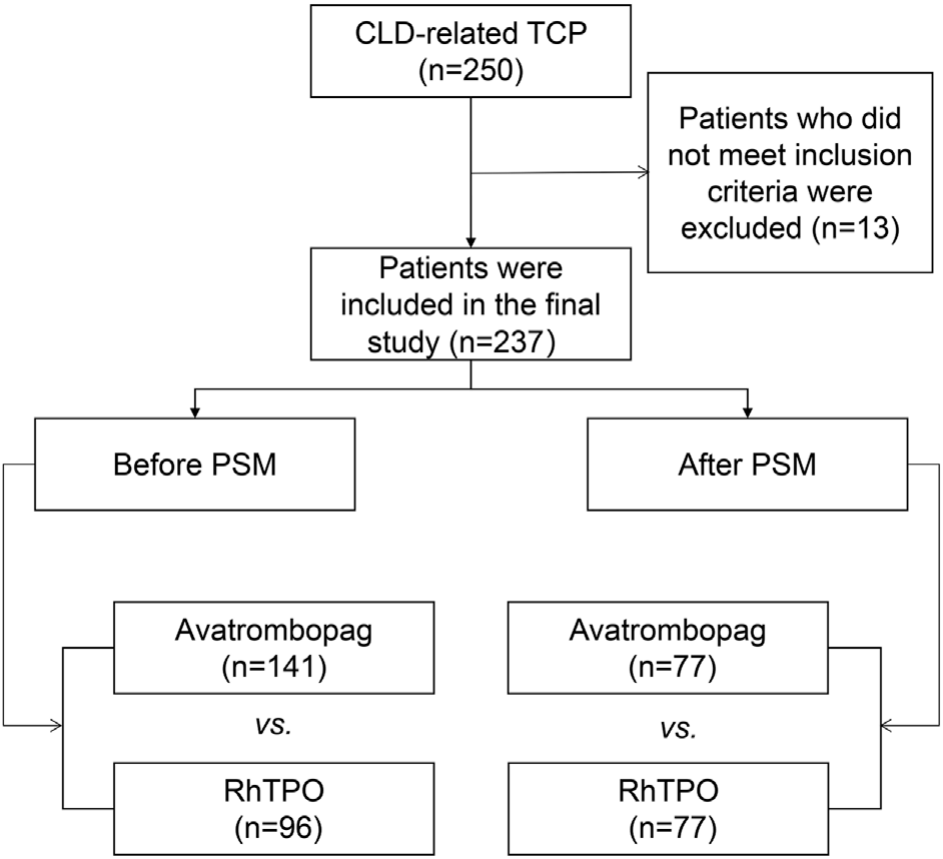
Flow chart of the study.

A total of 250 patients with TCP associated with CLD receiving avatrombopag or rh-TPO treatment were collected retrospectively in our hospital from January 2019 to January 2022. A total of 13 patients were excluded, and 237 patients were included based on the inclusion and exclusion criteria (Figure 1). The study protocol was approved by the Ethics Committee of the First Affiliated Hospital of the University of Science and Technology of China (ID: 2022-RE-016). The written informed consent form signed by the patient was exempted due to the retrospective nature of the study.

### 2.2 Inclusion and exclusion criteria

Inclusion criteria: 1) TCP associated with CLD, 2) treatment with avatrombopag or rh-TPO, and 3) complete clinical data.

Exclusion criteria: 1) combined with malignant hematological diseases, such as lymphoma and leukemia, 2) combined with major cardiovascular disease, 3) receiving other PLT-containing therapy simultaneously, and 4) missing clinical data (Terrault et al., 2018).

### 2.3 Therapeutic strategy

Treatment criteria: CLD patients with severe TCP need to have a platelet count above 50 × 10^9^/L before invasive surgical procedures are performed to reduce the risk of bleeding.

Patients in the avatrombopag treatment group: PLT <40 × 10^9^/L, 60 mg/d once per day and PLT from 40 to 50 × 10^9^/L, 40 mg/d once a day for 5 days orally. Patients in the rh-TPO treatment group received a subcutaneous dose of 300 U/(kg·d) once a day and continuous treatment for 1–5 days.

### 2.4 Observation and outcome indicators

Parameters assessed: 1) PLT change after using drugs: mean PLT counts after 1–5 days, 6–10 days, and 11–15 days of medication and 2) adverse drug events: portal vein thrombosis, headache, dizziness, blood transfusion reaction, hematuria, proteinuria, fever, abdominal pain, diarrhea, indigestion, fatigue, nausea, and peripheral tissue edema.

Outcome indicators: reaching the increased level of PLT counts ≥50 × 10^9^/L on the 15th day of evaluation after drug administration and no PLT infusion or no occurrence of emergency bleeding risk (Terrault et al., 2018). Therapeutic response rate equaled the number of patients with an effective endpoint/the number of patients included in each group ×100%.

### 2.5 Statistical analysis

SPSS statistical software (version 26.0) was used for the data analysis. The baseline population was described as the mean ± standard deviation (SD) or median (min, max). Anderson–Darling normality test was used for the normal distribution test. Normal distribution data were compared between the two groups by a *t-*test. Skew distribution data were compared between the two groups by the Mann–Whitney *U* test. Count data are expressed as absolute numbers or percentages and were compared between groups using the χ^2^ test or Fisher’s exact probability method. Repeated measurement data in-point comparisons between groups were done using generalized estimator equations. ROC diagnostic curves were used for platelet count before treatment and outcome indicators. *p* < 0.05 was considered statistically significant. The propensity score matching (PSM) method was used to adjust the baseline variables between the avatrombopag and rh-TPO groups to draw more accurate conclusions and avoid possible selection bias. Multivariate logistic regression analysis was used to determine the propensity score for each patient based on sex, age, body mass index (BMI), premedication PLT, pathological diagnosis, chronic hepatitis B infection, Child‒Pugh grade, and types of surgical procedure, with 1:1 matching and propensity score by nearest neighbor matching. After PSM, 77 patients were included in each group, and the detailed clinical parameters are summarized in Table 1.

**TABLE 1:** Baseline characteristics before and after propensity score matching (PSM).

Patient characteristic	Before PSM			After PSM		
	Avatrombopag (*n* = 141)	Rh-TPO (*n* = 96)	*P*	Avatrombopag (*n* = 77)	Rh-TPO (*n* = 77)	*P*
Sex, n (%)			**0.001**			0.595
Male	111 (78.7%)	57 (59.4%)		53 (68.8%)	56 (72.7%)	
Female	30 (21.3%)	39 (40.6%)		24 (31.2%)	21 (27.3%)	
Mean Age (±SD), years	56.6 (±11.4)	58.0 (±11.3)	0.368	57.4 (±12.0)	57.8 (±11.0)	0.823
Mean BMI (±SD), kg/m^2^	22.6 (±3.8)	21.7 (±3.0)	**0.046**	22.3 (±3.1)	22.0 (±3.1)	0.499
Median platelet count (mix, max), ×10^9^/L	34.0 (1,50)	29.5 (1,50)	**0.004**	33.0 (1,50)	32.0 (2,50)	0.736
Disease etiology			0.651			0.868
Cirrhosis	57 (40.4%)	36 (37.5%)		29 (37.7%)	30 (39.0%)	
Hepatoma	84 (59.6%)	60 (62.5%)		48 (62.3%)	47 (61.0%)	
Chronic hepatitis B infection, n (%)	97 (68.8%)	60 (62.5%)	0.314	53 (68.8%)	49 (63.6%)	0.496
Splenomegaly, n (%)	99 (70.2%)	76 (79.2%)	0.124	54 (70.1%)	58 (75.3%)	0.469
Child‒Turcotte–Pugh Class, n (%)			0.423			0.932
A	54 (38.3%)	41 (42.7%)		36 (46.8%)	34 (44.2%)	
B	47 (33.3%)	35 (36.5%)		23 (29.9%)	25 (32.5%)	
C	40 (28.4%)	20 (20.8%)		18 (23.4%)	18 (23.4%)	
Invasive surgical procedures			0.458			0.661
TACE	40 (28.4%)	27 (28.1%)		17 (22.1%)	20 (26.0%)	
Abdominal paracentesis	25 (17.7%)	9 (9.4%)		13 (16.9%)	7 (9.1%)	
Microwave ablation	6 (4.3%)	4 (4.2%)		6 (7.8%)	4 (5.2%)	
Laparoscopic hepatectomy	8 (5.7%)	4 (4.2%)		4 (5.2%)	4 (5.2%)	
Liver transplantation	4 (2.8%)	2 (2.1%)		2 (2.6%)	1 (1.3%)	
Others^a^	58 (41.1%)	50 (52.1%)		35 (45.5%)	41 (53.2%)	

Others^a^: lumbar puncture; endoscopic esophageal gastric varices injection; intravenous immunotherapy; conization of the cervix; implantation of iodine-125 radioactive particles; percutaneous transhepatic cholangio drainage (PTCD); transjugular intrahepatic portosystemic shunt (TIPS); catheterization of the right femoral vein; closed thoracic drainage; tension-free repair of inguinal hernia and no invasive operations. BMI (body mass index) and TACE (transcatheter arterial chemoembolization).

The meaning of bold values are statistical difference between the two groups (*p*<0.05).

## 3 Results

### 3.1 Baseline characteristics

Before PSM, characteristics including sex, age, BMI (kg/m^2^), mean PLT count before medication, disease type, chronic hepatitis B infection, splenomegaly, Child–Turcotte–Pugh Class grade, and type of invasive surgical procedures were compared between the avatrombopag and rh-TPO groups. There were significant differences in sex, BMI, and mean platelet count between the two groups before medication (*p* < 0.05). After PSM, there were no significant statistical differences in any of the abovementioned characteristics between the two groups (*p* > 0.05). The two groups were comparable (Table 1).

### 3.2 Before propensity score matching

#### 3.2.1 Platelet count changes and therapeutic response rate

**FIGURE 2:**
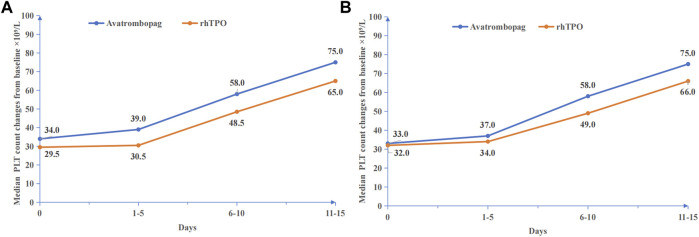
Platelet count changes before and after medication. **(A)** Before PSM, median PLT count changes from baseline ×10^9^/L. **(B)** After PSM, median PLT count changes from baseline ×10^9^/L.

The median changes in platelet count before and after treatment in the avatrombopag and rh-TPO groups were compared (Figure 2A). The effective rate was 75.18% (106/141) in the avatrombopag group and 68.75% (66/96) in the rh-TPO group, and there was no statistical difference between the two groups (*p* = 0.276).

In patients with ineffective response, six patients received platelet transfusions in the avatrombopag group and eight patients received platelet transfusions in the rh-TPO group. There was no significant difference in the proportion of patients receiving platelet transfusion between the two groups (*p* = 0.191).

The PLT count showed a time-dependent increase in the treatment duration of both drugs (*Waldχ* ^*2*^ = 176.413, *p* < 0.001). However, no statistically significant difference between the avatrombopag group and the rh-TPO group in the treatment of severe TCP related to CLD was observed with the increased PLT count (*Waldχ* ^*2*^ = 1.659, *p* = 0.198). In addition, no interaction between time and different medications was found (*Waldχ* ^*2*^ = 0.540, *p* = 0.910) (Figure 2A).

#### 3.2.2 Stratification of the two groups according to Child–Turcotte–Pugh Class grade and comparison of the drug efficacy of the two groups

Before PSM (Table 2), in Child‒Pugh Class A, the effective rate was 88.89% in the avatrombopag group and 63.41% in the rh-TPO group. There was a significant difference in the effective rate between the two groups (*p* = 0.003). In Child‒Pugh Class B, the effective rate was 76.60% in the avatrombopag group and 72.00% in the rh-TPO group. There was no significant difference in the effective rate between the two groups (*p* = 0.417). In addition, in Child‒Pugh Class C, the effective rate was 55.00% in the avatrombopag group and 80.00% in the rh-TPO group. There was no significant difference in the effective rate between the two groups (*p* = 0.088).

**TABLE 2:** Effective rate of drug therapy according to Child–Pugh Class.

		Before PSM				After PSM			
		Efficient	Total	*χ* ^*2*^	*P*	Efficient	Total	*χ* ^*2*^	*P*
Child‒Pugh	Avatrombopag	48 (88.89%)	54 (100%)	8.783	**0.003**	30 (83.33%)	36 (100%)	4.113	**0.043**
Class A	Rh-TPO	26 (63.41%)	41 (100%)			21 (61.76%)	34 (100%)		
Child‒Pugh	Avatrombopag	36 (76.60%)	47 (100%)	0.658	0.417	16 (69.57%)	23 (100%)	0.034	0.853
Class B	Rh-TPO	18 (72.00%)	25 (100%)			18 (72.00%)	25 (100%)		
Child‒Pugh	Avatrombopag	22 (55.00%)	40 (100%)	—	0.088^a^	12 (66.67%)	18 (100%)	—	0.711^a^
Class C	Rh-TPO	16 (80.00%)	20 (100%)			14 (77.78%)	18 (100%)		

Note: ^a^ adopts Fisher’s exact probability test.

The meaning of bold values are statistical difference between the two groups (*p*<0.05).

#### 3.2.3 Stratification of the two groups according to the baseline level of platelet and comparison of the drug efficacy of the two groups

**FIGURE 3:**
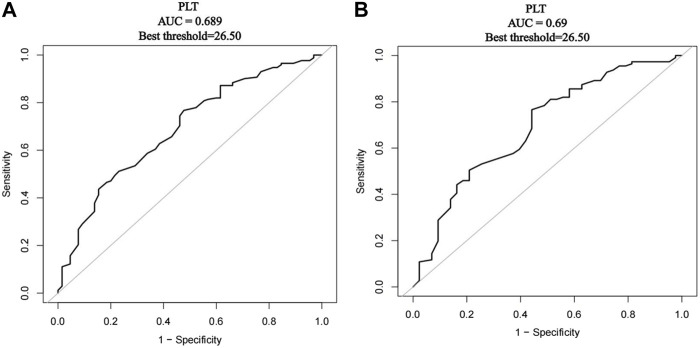
Receiver operating characteristics (ROC) curve of platelet count diagnosed with outcome indicators. **(A)** Before PSM, the ROC diagnostic curve of platelet count diagnosed with outcome indicators. **(B)** After PSM, the ROC diagnostic curve of platelet count diagnosed with outcome indicators.

Regardless of whether before or after PSM, the best threshold of PLT was 26.5 × 10^9^/L by the ROC diagnosis curve (Figures 3A and B). Thus, according to the baseline level of platelets, they were divided into PLT ≤25 × 10^9^/L and 26–50 × 10^9^/L to compare the drug effectiveness of the two groups (Table 3). When PLT ≤25 × 10^9^/L, the effective rate was 55.17% in the avatrombopag group and 53.85% in the rh-TPO group. There was no significant difference in effective rate between the two groups (*p* = 0.914). When PLT ranged from 26 to 50 × 10^9^/L, the effective rate was 80.36% in the avatrombopag group and 78.95% in the rh-TPO group. There was no significant difference in effective rate between the two groups (*p* = 0.829). However, the efficiency of the avatrombopag group with PLT ≤25 × 10^9^/L was significantly lower than that with PLT ranging from 26 ×1 0^9^/L to 50 × 10^9^/L (55.17% vs*.* 80.36%). Moreover, the efficiency of the rh-TPO group was significantly lower when PLT ≤25 × 10^9^/L than when PLT ranged from 26 to 50 × 10^9^/L (53.85% vs. 78.95%).

**TABLE 3:** Effective rate of drug therapy according to different platelet baselines.

		Before PSM				After PSM			
		Efficient	Total	*χ* ^*2*^	*P*	Efficient	Total	*χ* ^*2*^	*P*
PLT (≤25 × 10^9^/L)	Avatrombopag	16 (55.17%)	29 (100%)	0.012	0.914	10 (52.63%)	19 (100%)	0.027	0.958
	Rh-TPO	21 (53.85%)	39 (100%)			14 (51.85%)	27 (100%)		
PLT (26–50 × 10^9^/L)	Avatrombopag	90 (80.36%)	112 (100%)	0.047	0.829	48 (82.76%)	58 (100%)	0.384	0.144
	Rh-TPO	45 (78.95%)	57 (100%)			39 (78.00%)	50 (100%)		

#### 3.2.4 Drug treatment-related adverse events

Before PSM (Table 4), except for the incidence of dizziness, there was no significant difference in the incidence of adverse drug events, including portal vein thrombosis, abdominal pain, fatigue, indigestion, headache, nausea, diarrhea, and peripheral edema, between these two groups (*p* > 0.05). Fewer patients reported dizziness and pyrexia in the avatrombopag group than in the rh-TPO group (7.8% vs. 25.0%, 9.2% vs. 21.9%, both *p* < 0.05). In addition, there were no serious bleeding events in the short term after medication.

**TABLE 4:** Adverse events.

	Before PSM			After PSM		
	Avatrombopag	Rh-TPO	*P*	Avatrombopag	Rh-TPO	*P*
Portal vein thrombosis	4 (2.8%)	4 (4.2%)	0.578^a^	2 (2.6%)	2 (2.6%)	1.000^a^
Abdominal pain	32 (22.7%)	16 (16.7%)	0.257	16 (20.8%)	11 (14.3%)	0.289
Nausea	21 (14.9%)	18 (18.8%)	0.432	11 (14.3%)	14 (18.2%)	0.381
Fatigue	28 (19.9%)	24 (25.0%)	0.348	11 (14.3%)	20 (26.0%)	0.070
Pyrexia	13 (9.2%)	21 (21.9%)	**0.006**	7 (9.1%)	14 (18.2%)	0.100
Indigestion	20 (14.2%)	17 (17.7%)	0.463	7 (9.1%)	9 (11.7%)	0.612
Dizziness	11 (7.8%)	24 (25.0%)	**0.001**	6 (7.8%)	19 (24.7%)	**0.005**
Headache	9 (6.4%)	6 (6.2%)	0.967	5 (6.5%)	5 (6.5%)	1.000
Diarrhea	13 (9.2%)	13 (13.5%)	0.296	5 (6.5%)	5 (6.5%)	1.000
Peripheral edema	9 (6.4%)	9 (9.4%)	0.393	5 (6.5%)	9 (11.7%)	0.262

Note: ^a^ adopts Fisher’s exact probability test.

The meaning of bold values are statistical difference between the two groups (*p*<0.05).

### 3.3 After propensity score matching

#### 3.3.1 Platelet count changes and therapeutic response rate

The median changes in PLT count before and after treatment in the avatrombopag and rh-TPO groups are shown in Figure 2B. The effective rate was 75.32% (58/77) in the avatrombopag group and 68.83% (53/77) in the rh-TPO group, and there was no statistical difference between the two groups (*p* = 0.369).

In patients with ineffective response, three patients received platelet transfusions in the avatrombopag group and four patients received platelet transfusions in the rh-TPO group. There was no significant difference in the proportion of patients receiving platelet transfusion between the two groups (*p* = 1.000).

The PLT count showed a time-dependent increase in the treatment duration of both drugs (*Waldχ* ^*2*^ = 142.210, *p* < 0.001). However, no statistically significant difference between the avatrombopag group and the rh-TPO group in the treatment of severe TCP related to CLD was observed in the effect of increasing PLT count (*Waldχ* ^*2*^ = 0.220, *p* = 0.639). In addition, no interaction between time and different medications was found (*Waldχ* ^*2*^ = 1.273, *p* = 0.736) (Figure 2B).

#### 3.3.2 Stratification of the two groups according to Child–Turcotte–Pugh Class grade and comparison of the drug efficacy of the two groups

After PSM (Table 2), in Child–Pugh Class A, the effective rate was 83.33% in the avatrombopag group and 61.76% in the rh-TPO group. There was a significant difference in the effective rate between the two groups (*p* = 0.043). In Child‒Pugh Class B, the effective rate was 69.57% in the avatrombopag group and 72.00% in the rh-TPO group. There was no significant difference in the effective rate between the two groups (*p* = 0.853). In Child‒Pugh Class C, the effective rate was 66.67% in the avatrombopag group and 77.78% in the rh-TPO group. There was no significant difference in the effective rate between the two groups (*p* = 0.711).

#### 3.3.3 Stratification of the two groups according to the baseline level of platelet and comparison of the drug efficacy of the two groups

After PSM, according to the baseline level of platelets, they were also divided into PLT ≤25 × 10^9^/L and 26–50 × 10^9^/L to compare the drug effectiveness of the two groups (Table 3). When PLT ≤25 × 10^9^/L, the effective rate was 52.63% in the avatrombopag group and 51.85% in the rh-TPO group. There was no significant difference in effective rate between the two groups (*p* = 0.911). When PLT ranged from 26 to 50 × 10^9^/L, the effective rate was 82.76% in the avatrombopag group and 78.00% in the rh-TPO group. There was no significant difference in the effective rate between the two groups (*p* = 0.958). However, the efficiency of the avatrombopag group with PLT ≤25 × 10^9^/L was significantly lower than that with PLT ranging from 26 × 10^9^/L to 50 × 10^9^/L (52.63% vs*.* 82.76%). Moreover, the efficiency of the rh-TPO group was significantly lower when PLT ≤25 × 10^9^/L than when PLT ranged from 26 to 50 × 10^9^/L (51.85% *vs.* 78.00%).

#### 3.3.4 Drug treatment-related adverse events

After PSM (Table 4), except for the incidence of dizziness, there was no significant difference in the incidence of adverse drug events, including portal vein thrombosis, abdominal pain, pyrexia, fatigue, indigestion, headache, nausea, diarrhea, and peripheral edema, between these two groups (*p* > 0.05). Fewer patients reported dizziness in the avatrombopag group than in the rh-TPO group (7.8% vs*.* 24.7%, *p* = 0.005). In addition, there were no serious bleeding events in the short term after medication.

## 4 Discussion

At present, rh-TPO, glucocorticoids, avatrombopag, and lusutrombopag are commonly used in the clinical treatment of CLD-related TCP. Rh-TPO is a full-length glycosylated TPO purified by gene recombination technology. Rh-TPO and endogenous TPO have similar pharmacological effects in increasing PLT by activating C-MPL (Demetri, 2000). However, problems with the clinical use of rh-TPO include subcutaneous administration and an inability to predict an increase in PLT count. Therefore, new improved drugs or alternative drugs are constantly being explored and developed. Avatrombopag is a novel, oral, small-molecule TPO-R agonist (Terrault et al., 2014; Terrault et al., 2018). It ultimately promotes megakaryocyte proliferation/differentiation and predictably increases the PLT count through the activation of the JAK-STAT and SHC-Ras-Raf-ERK signaling pathways and, therefore, could be used as an alternative to PLT infusion (Bussel et al., 2014; Kuter and Allen, 2018; Długosz-Danecka et al., 2019). Avatrombopag only increases the number of PLT in patients and does not enhance PLT activation (Michelson et al., 2018; Kuter, 2022). To date, there have been few real-world studies of these two drugs in the treatment of TCP associated with CLD, let alone any comparisons between the two drugs.

Two global, multicenter, randomized, double-blind, placebo-controlled phase II (E5501-G000-202, NCT00914927) and phase III clinical trials (ADAPT-1, ADAPT-2) evaluated the efficacy and safety of avatrombopag in patients with severe TCP related to CLD who underwent invasive procedures or surgery. The results suggest that avatrombopag can be used to treat severe TCP related to CLD with high efficacy and safety (Terrault et al., 2014; Terrault et al., 2018). A nationwide multicenter, real-world study has reported the efficacy and safety of rh-TPO in the treatment of TCP caused by cirrhosis (Feng et al., 2022). At the same time, an ethnic sensitivity analysis concluded that avatrombopag was effective and safe in the management of thrombocytopenia in Chinese patients with CLD. Ethnicity does not appear to influence the efficacy and safety of avatrombopag (Lu et al., 2022). Practice Guidelines of the Central European Hepatologic Collaboration (CEHC) on the use of TPO receptor agonists in patients with CLD undergoing invasive procedures were reported. A consensus was agreed that TPO-RAs should be considered for raising PLT count in CLD patients undergoing scheduled abdominal surgery, high-bleeding risk dentistry, endoscopic polypectomy, endoscopic variceal ligation, liver biopsy, liver surgery, liver transplantation, and percutaneous ablation, but it was also agreed that they are less beneficial or not necessary for endoscopy without intervention and paracentesis (Flisiak et al., 2021). Our study included patients with these high-risk procedures, and there were no serious bleeding events in the short term after medication.

In the present real-world study, two important results were found. First, the treatment response rate was 75.18% (106/141) in the avatrombopag group and 68.75% (66/96) in the rh-TPO group. Both before and after PSM, there was no statistically significant difference in treatment efficiency between the two groups. However, in the treatment of Child‒Pugh Class A patients both before and after PSM, the effective rate of the avatrombopag group was higher than that of the rh-TPO group, with a statistically significant difference between the two groups (88.89% vs. 63.41%; 81.33% vs. 61.76%). The use of avatrombopag in the treatment of TCP associated with CLD in Child‒Pugh Class A may achieve better clinical efficacy and significantly reduce the risk of invasive procedures. We thought that this might be because of the way in which it was administered: avatrombopag is administered orally, while rh-TPO is administered subcutaneously. In patients with better liver function, the more effective the drug is absorbed through the digestive system, the better the platelet count will be. For patients with severe liver disease, oral medication is far less effective than injection (Pereira et al., 2005). The clinical response rate of avatrombopag was lower than that of the two phase III clinical trials (75.18% vs. 88.1%/87.9%), which may be due to the difference in the patient populations selected: few patients with liver function in the stage of Child‒Pugh Class C were included, and patients with hepatocellular carcinoma (Barcelona-Clinic Liver Cancer staging classification C or D) were excluded from these clinical trials (Terrault et al., 2014; Terrault et al., 2018). Given the actual clinical situation, these patients excluded from prospective clinical trials will also have application scenarios of the abovementioned drugs in the real world. Second, the efficacy rates of avatrombopag and rh-TPO in patients with initial PLTs >25 × 10^9^/L were higher than those in patients with initial PLTs ≤25 × 10^9^/L both before and after PSM (55.17% vs. 80.36%, 53.85% vs. 78.95%; 55.56% vs. 81.36%, 53.85% vs. 76.47%). Considering the results of ROC analysis (the best threshold of PLT was 26.5 × 10^9^/L both before and after PSM) and the convenience for clinical use, we set the cutoff value as 25 × 10^9^/L.

Regarding safety, there were no serious systemic adverse events caused in the two groups. The most common complications were abdominal pain, fatigue, dizziness, and fever. There was no significant difference in the incidence of abdominal pain, pyrexia, fatigue, indigestion, nausea, diarrhea, peripheral edema, or other adverse events between the two groups. However, the incidence of dizziness in the rh-TPO group was higher than that in the avatrombopag group both before and after PSM (25.0% vs. 7.8%; 24.7% vs. 7.8%). The incidence of abdominal pain and fatigue was higher than that in clinical trials (22.75 vs. 10.3%; 19.9% vs. 6.7%), and there were no significant differences in other adverse events. This may be because we included more patients with Child‒Pugh Class C, whereas clinical trials rarely included such patients (Terrault et al., 2018). These patients have abdominal pain and fatigue symptoms, and it can be difficult to distinguish whether the drug causes these symptoms. In addition, with respect to the delivery route, patients treated with rh-TPO require subcutaneous injection, while patients treated with avatrombopag require oral administration, which is more convenient and safer.

There are still some limitations in our study. First, compared with the prospective clinical trials, it is difficult to monitor patients’ PLT counts dynamically each day due to various issues such as ethics and patient compliance in a real-world study. Second, the change in PLT counts was only directly observed up to 14 days after medication due to the urgency and need for surgical treatment of the patient’s disease, especially in cases where the PLT counts met the minimum requirement for invasive surgery or related treatment (>50 × 10^9^/L). Thus, a long-term result of efficacy and safety needs to be observed in future studies if conditions permit. Third, a limited number of cases were included in the study due to the short time that avatrombopag has been on the market. All of these issues will be focused on and updated further in our future studies.

## 5 Conclusion

Taken together, to the best of our knowledge, this is the first real-world study focusing on the comparison of efficacy and safety between avatrombopag and rh-TPO. Our findings proved that avatrombopag showed better clinical efficacy than rh-TPO in the treatment of TCP associated with CLD in Child‒Pugh Class A and showed a lower incidence of dizziness in all patients. The effective rates of avatrombopag and rh-TPO in patients with initial PLTs >25 × 10^9^/L were higher than those in patients with initial PLTs ≤25 × 10^9^/L. In the future, a large cohort or prospective clinical study still needs to be performed to further verify the abovementioned findings.

## Data Availability

The raw data supporting the conclusion of this article will be made available by the authors, without undue reservation.

## Abbreviations

TCP, thrombocytopenia; CLD, chronic liver disease; rh-TPO, recombinant human thrombopoietin; PLT, platelet; PSM, propensity score matching; TPO, thrombopoietin; BMI, body mass index; TACE, transcatheter arterial chemoembolization.
